# Differences in optic nerve head structure between acute angle-closure glaucoma and open-angle glaucoma

**DOI:** 10.1038/s41598-023-35020-y

**Published:** 2023-05-16

**Authors:** Jeong Han Kong, Sung Pyo Park, Kyeong Ik Na

**Affiliations:** Department of Ophthalmology, Kangdong Sacred Heart Hospital, 150, Seongan-ro, Gangdong-gu, Seoul, 05355 South Korea

**Keywords:** Diseases, Medical research

## Abstract

This study aimed to compare the optic nerve head (ONH) structure in acute angle-closure glaucoma (AACG) and open-angle glaucoma (OAG) to investigate the differences in glaucomatous damage. The AACG and OAG eyes were matched with regard to global retinal nerve fiber layer thickness (RNFLT). AACG eyes were divided into two subgroups based on the presence of ONH swelling at the onset of AACG. RNFLT, Bruch’s membrane opening-minimum rim width (BMO-MRW), and Bruch’s membrane opening-minimum rim area (BMO-MRA) were analyzed. Global RNFLT values were similar in AACG and OAG groups, but lower than in the healthy group (*P* < 0.001). The global BMO-MRW and total BMO-MRA were significantly higher in AACG than in OAG group (*P* < 0.001, respectively). AACG showed similar global BMO-MRW and total BMO-MRA, irrespective of the presence or absence of ONH swelling, while AACG with ONH swelling was associated with significantly thinner global RNFLT compared to AACG without ONH swelling (*P* < 0.006). The result of differences in ONH structure between the OAG and AACG, especially the AACG with ONH swelling at the onset of AACG, suggests that the mechanisms of optic nerve damage in the two diseases are different.

## Introduction

Acute angle-closure (AAC) is a potentially blinding ophthalmic emergency caused by a sudden increase in intraocular pressure (IOP) due to obstruction of the anterior chamber angle. AAC can lead to irreversible damage to the optic nerve, which is diagnosed as acute angle-closure glaucoma (AACG)^1^. In contrast to AACG, open-angle glaucoma (OAG) is a chronic disease, in which the optic nerve head (ONH) is structurally changed due to the influence of intraocular pressure above the level that the optic nerve can tolerate.

Previously, there have been reports that ONH swelling was observed at the onset of AACG^2–6^. ONH edema is generally caused by inflammation or ischemia of the ONH and is observed in optic neuritis or non-arteritic anterior ischemic optic neuropathy^7,8^. However, it is rare in the course of OAG. This means that the mechanism of the optic nerve damage due to the abrupt increase in intraocular pressure in AACG may be different from that in OAG. This difference in the mechanism might lead to differences in the morphological features of ONH between AACG and OAG.

Hence, we compared the structure of the ONH in AACG and OAG using Bruch’s membrane opening (BMO)-based ONH parameters to improve the understanding of the optic nerve damage in the two types of glaucoma.

## Results

Eight out of the 38 eyes were excluded from the study because they had spectral domain-optical coherence tomography (SD-OCT) segmentation errors in the retinal nerve fiber layer (RNFL) and ONH analysis. Upon matching the 30 AACG eyes with OAG and healthy group, a total of 90 eyes (30 eyes selected from each of the OAG and healthy groups) were included. Demographic characteristics are summarized in Table 1. There were no significant differences in age, sex, or IOP among the three groups.

**Table 1:** Comparison of demographics and peripapillary retinal nerve fiber layer thickness (RNFLT) among the three subject groups.

	Healthy (n = 30)	OAG (n = 30)	AACG (n = 30)	*P* value	Post hoc
Age, years	66.53 ± 7.74	66.60 ± 6.10	68.17 ± 7.87	0.618*	
Male, n (%)	14 (46.6)	13 (43.3)	14 (46.6)	0.958†	
IOP	12.87 ± 2.89	12.07 ± 2.00	12.70 ± 5.03	0.655*	
Global RNFL (µm)	101.07 ± 9.45	82.43 ± 9.28	82.57 ± 22.35	< 0.001*	Healthy > OAG, AACG
Superior temporal RNFLT (µm)	130.10 ± 19.52	100.70 ± 26.19	100.63 ± 33.82	< 0.001*	Healthy > OAG, AACG
Temporal RNFLT (µm)	74.10 ± 16.11	65.90 ± 15.91	71.37 ± 16.15	0.138*	
Inferior temporal RNFLT (µm)	153.40 ± 13.73	98.77 ± 44.27	118.80 ± 44.45	< 0.001*	Healthy > AACG > OAG
Inferior nasal RNFLT (µm)	110.67 ± 19.63	83.03 ± 20.87	92.67 ± 28.86	< 0.001*	Healthy > OAG, AACG
Nasal RNFLT (µm)	80.47 ± 11.72	71.13 ± 8.98	65.93 ± 20.71	0.001*	Healthy > OAG, AACG
Superior nasal RNFLT (µm)	127.57 ± 24.08	102.47 ± 22.53	89.83 ± 36.16	< 0.001*	Healthy > OAG, AACG

Continuous variables are presented as mean ± SD.

*OAG* open-angle glaucoma, *AACG* acute angle-closure glaucoma, *IOP* intraocular pressure.

*One-way analysis of variance with Duncan’s post hoc test.

†Chi-square test.

**Figure 1:**
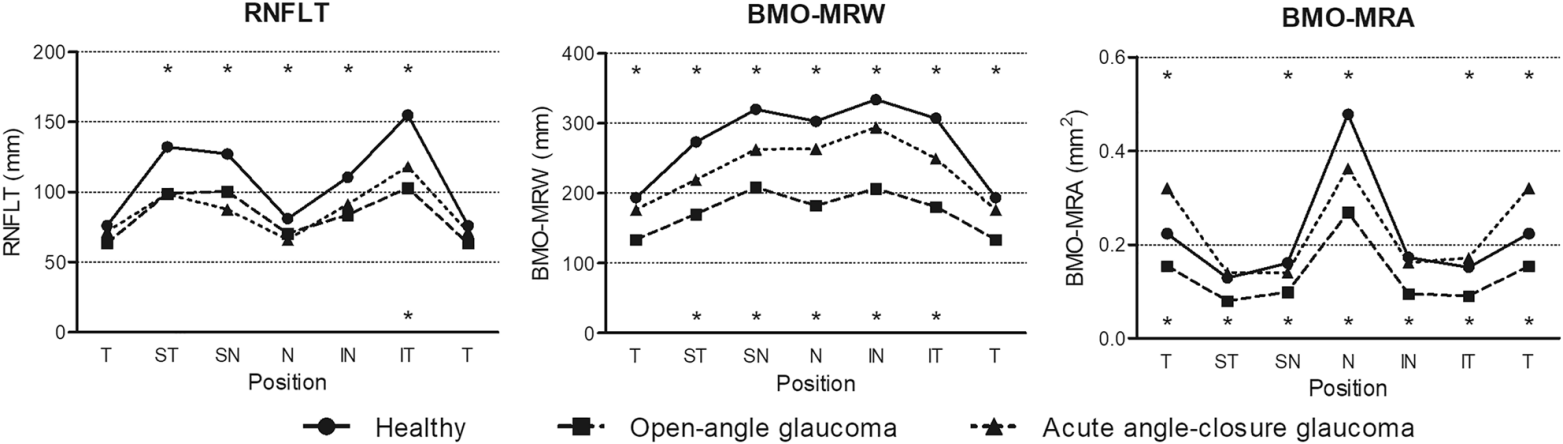
Comparison of peripapillary retinal nerve fiber layer thickness (RNFLT), Bruch’s membrane opening-minimum rim width (BMO-MRW), and Bruch’s membrane opening-minimum rim area (BMO-MRA) in each sector among the three subject groups. For the sectors marked with an asterisk above the dots, the one-way analysis of variance with Duncan’s post hoc test revealed a significant difference (*P* < 0.05) between the two higher values; in contrast, for the sectors marked with an asterisk below the dots, a significant difference (*P* < 0.05) was found between the two lower.

RNFL thickness (RNFLT) was significantly lower in the AACG and OAG groups than that in the healthy group in the global and all sectors except the temporal sector. The RNFLT was not different between the AACG and OAG groups, except in the inferior temporal sector (Table 1, Fig. 1), where it was lower in the OAG group than in the AACG group (*P* < 0.001).

The BMO area was matched among the three groups, and there was no significant difference between them (*P* = 0.996) (Table 2). Bruch’s membrane opening-minimum rim width (BMO-MRW) was highest in the healthy group, followed by the AACG and OAG groups in the global and all sectors, except the temporal sector (all *P* < 0.001) (Table 2, Fig. 1). In the temporal sector, BMO-MRW was not different between the healthy and OAG groups but was higher than that of the OAG group (*P* < 0.001).

**Table 2:** Comparison of optic nerve head parameters among the three subject groups.

	Healthy (n = 30)	OAG (n = 30)	AACG (n = 30)	*P* value*	Post hoc
BMO area	2.19 ± 0.44	2.18 ± 0.37	2.19 ± 0.50	0.996	
BMO-MRW					
Global BMO-MRW (µm)	276.80 ± 35.53	177.40 ± 27.27	239.13 ± 55.44	< 0.001	Healthy > AACG > OAG
Superior temporal BMO-MRW (µm)	271.17 ± 41.95	176.60 ± 50.76	222.20 ± 68.04	< 0.001	Healthy > AACG > OAG
Temporal BMO-MRW (µm)	191.77 ± 37.51	135.73 ± 30.67	178.53 ± 39.18	< 0.001	Healthy, AACG > OAG
Inferior temporal BMO-MRW (µm)	302.57 ± 35.78	179.13 ± 59.45	249.53 ± 82.44	< 0.001	Healthy > AACG > OAG
Inferior nasal BMO-MRW (µm)	336.50 ± 49.09	205.37 ± 55.63	295.47 ± 78.03	< 0.001	Healthy > AACG > OAG
Nasal BMO-MRW (µm)	302.37 ± 45.72	186.83 ± 35.89	261.10 ± 63.52	< 0.001	Healthy > AACG > OAG
Superior nasal BMO-MRW (µm)	314.53 ± 60.78	215.43 ± 50.14	266.53 ± 81.00	< 0.001	Healthy > AACG > OAG
BMO-MRA					
Total BMO-MRA (mm^2^)	1.301 ± 0.238	0.762 ± 0.138	1.290 ± 0.229	< 0.001	Healthy, AACG > OAG
Superior temporal BMO-MRA (mm^2^)	0.129 ± 0.028	0.079 ± 0.020	0.136 ± 0.035	< 0.001	Healthy, AACG > OAG
Temporal BMO-MRA (mm^2^)	0.223 ± 0.050	0.149 ± 0.039	0.310 ± 0.098	< 0.001	AACG > Healthy > OAG
Inferior temporal BMO-MRA (mm^2^)	0.150 ± 0.027	0.087 ± 0.031	0.168 ± 0.037	< 0.001	AACG > Healthy > OAG
Inferior nasal BMO-MRA (mm^2^)	0.173 ± 0.038	0.090 ± 0.028	0.161 ± 0.032	< 0.001	Healthy, AACG > OAG
Nasal BMO-MRA (mm^2^)	0.482 ± 0.095	0.260 ± 0.053	0.375 ± 0.113	< 0.001	Healthy > AACG > OAG
Superior nasal BMO-MRA (mm^2^)	0.158 ± 0.036	0.097 ± 0.026	0.140 ± 0.031	< 0.001	Healthy > AACG > OAG

Values are presented as mean ± SD.

*OAG* open-angle glaucoma, *AACG* acute angle-closure glaucoma, *BMO* Bruch’s membrane opening, *MRW* minimum rim width, *MRA* minimum rim area.

*One-way analysis of variance with Duncan’s post hoc test.

Bruch’s membrane opening-minimum rim area (BMO-MRA) was lower in the OAG group than that of the other two groups in the total and all sectors (all *P* < 0.001) (Table 2, Fig. 1). In the total, superior temporal, and inferior nasal sectors, BMO-MRA did not differ between the healthy and OAG groups. In the temporal and inferior temporal sectors, the BMO-MRA was higher in the AACG group than that of the healthy group. In the nasal and superior nasal sectors, BMO-MRA was higher in the healthy group than that of the AACG group.

**Figure 2:**
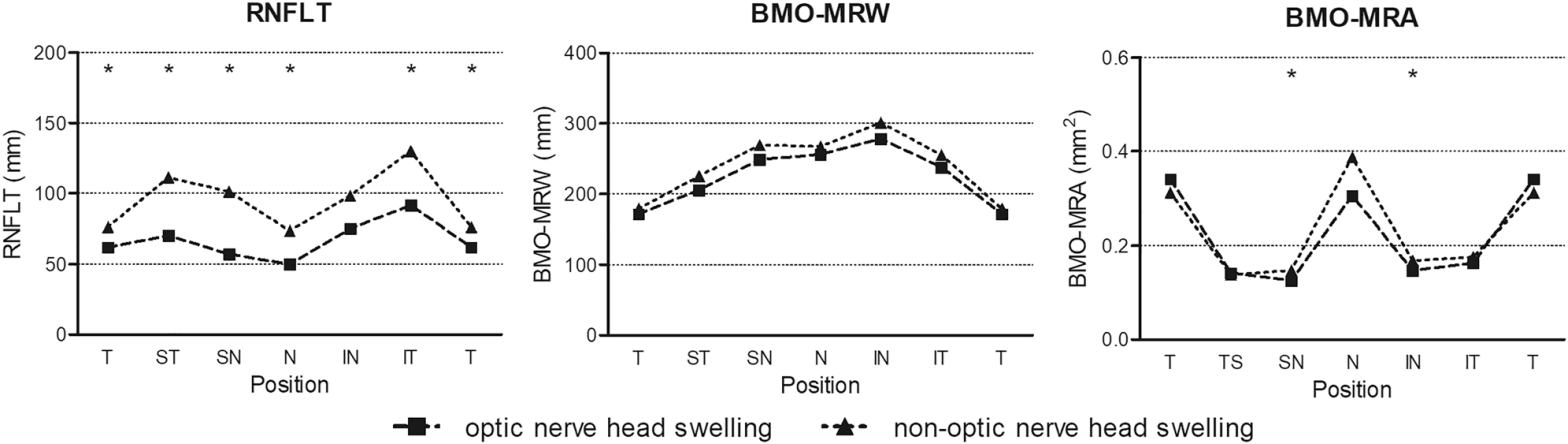
Comparison of peripapillary retinal nerve fiber layer (RNFLT), Bruch’s membrane opening-minimum rim width (BMO-MRW), and Bruch’s membrane opening-minimum rim area (BMO-MRA) in each sector among the two subgroups in acute angle-closure glaucoma. For the sectors marked with an asterisk above the dots, there is a significant difference (*P* < 0.05) in the Mann–Whitney test.

The AACG group was divided into ONH swelling AACG and non-ONH swelling AACG groups based on the presence of ONH swelling at AACG onset. RNFLT, BMO-MRW, and BMO-MRA results were compared between the two groups (Tables 3 and 4, Fig. 2). The RNFNT of the ONH swelling AACG group was significantly lower than that of the non-ONH swelling AACG group in the global and all sectors except the inferior nasal sector. However, there was no significant difference in BMO-MRW. BMO-MRA also did not differ between the ONH swelling AACG and non-ONH swelling AACG groups in all sectors, except the inferior and superior nasal sectors.

**Table 3:** Comparison of demographics and peripapillary retinal nerve fiber layer thickness (RNFLT) among the two subgroups of acute angle-closure glaucoma (AACG).

	ONH swelling AACG (n = 11)	Non-ONH swelling AACG (n = 19)	*P* value
Age, years	66.45 ± 7.90	69.16 ± 7.90	0.420*
Male, n (%)	4 (36.4)	9 (47.4)	0.708†
IOP (after attack)	10.64 ± 2.42	13.89 ± 5.78	0.185*
IOP (angle closure attack)	47.09 ± 11.61	47.74 ± 12.48	0.525*
Global RNFLT (µm)	68.45 ± 21.29	90.74 ± 18.99	0.006*
Superior temporal RNFLT (µm)	78.73 ± 33.59	113.32 ± 27.45	0.008*
Temporal RNFLT (µm)	63.09 ± 15.51	76.16 ± 14.84	0.023*
Inferior temporal RNFLT (µm)	97.00 ± 37.55	131.42 ± 44.06	0.021*
Inferior nasal RNFLT (µm)	81.73 ± 32.18	99.00 ± 25.51	0.077*
Nasal RNFLT (µm)	53.27 ± 18.18	73.26 ± 18.78	0.003*
Superior nasal RNFLT (µm)	70.82 ± 46.46	100.84 ± 23.64	0.023*

Continuous variables are presented as mean ± SD.

*ONH* optic nerve head.

*Mann–Whitney test.

^†^Chi-square test.

**Table 4:** Comparison of optic nerve head (ONH) parameters among the two subgroups in acute angle-closure glaucoma (AACG).

	ONH swelling AACG (n = 11)	non-ONH swelling AACG (n = 19)	*P* value*
BMO area	2.10 ± 0.51	2.24 ± 0.50	0.832
BMO-MRW			
Global BMO-MRW (µm)	230.09 ± 59.92	244.37 ± 53.65	0.328
Superior temporal BMO-MRW (µm)	215.36 ± 67.67	226.16 ± 69.79	0.641
Temporal BMO-MRW (µm)	174.55 ± 35.98	180.84 ± 41.70	0.641
Inferior temporal BMO-MRW (µm)	227.91 ± 91.78	262.05 ± 76.29	0.232
Inferior nasal BMO-MRW (µm)	275.82 ± 78.53	306.84 ± 77.54	0.185
Nasal BMO-MRW (µm)	253.09 ± 67.36	265.74 ± 62.59	0.471
Superior nasal BMO-MRW (µm)	262.82 ± 89.87	268.68 ± 77.92	0.641
BMO-MRA			
Total BMO-MRA (mm^2^)	1.217 ± 0.281	1.331 ± 0.189	0.103
Superior temporal BMO-MRA (mm^2^)	0.133 ± 0.042	0.138 ± 0.033	0.420
Temporal BMO-MRA (mm^2^)	0.317 ± 0.098	0.306 ± 0.100	0.767
Inferior temporal BMO-MRA (mm^2^)	0.155 ± 0.044	0.175 ± 0.031	0.112
Inferior nasal BMO-MRA (mm^2^)	0.147 ± 0.028	0.170 ± 0.032	0.026
Nasal BMO-MRA (mm^2^)	0.340 ± 0.102	0.395 ± 0.116	0.328
Superior nasal BMO-MRA (mm^2^)	0.125 ± 0.031	0.149 ± 0.027	0.033

Values are presented as mean ± SD.

*BMO* Bruch’s membrane opening, *MRW* minimum rim width, *MRA* minimum rim.

*Mann–Whitney test.

**Figure 3:**
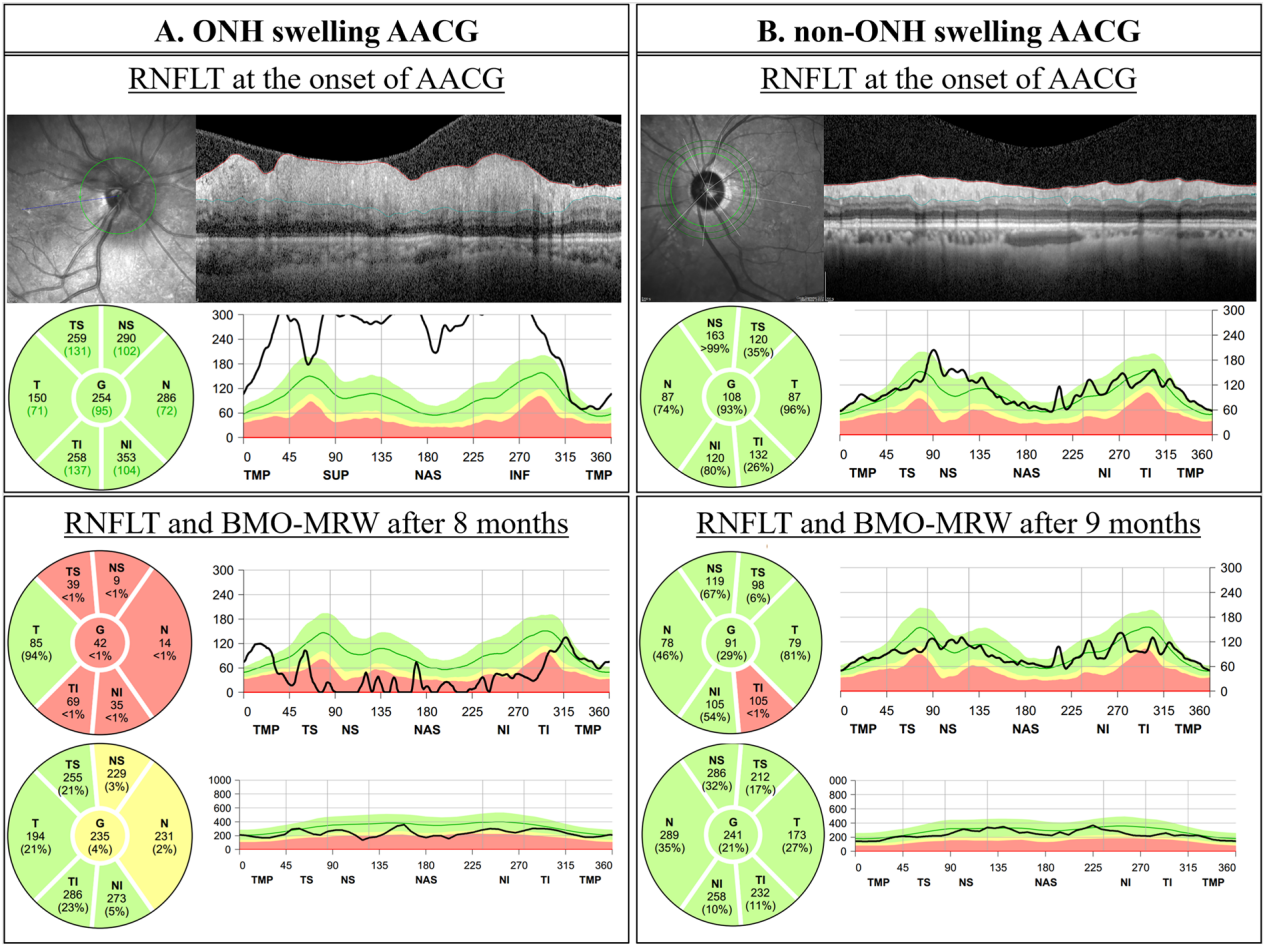
Representative cases of optic nerve head (ONH) swelling acute angle-closure glaucoma (AACG), and non-ONH swelling AACG groups. ONH swelling AACG eye, which shows retinal nerve fiber layer thickness (RNFLT) swelling at the onset of AACG, shows no difference in Bruch’s membrane opening-minimum rim width (BMO-MRW) compared to that of the non-ONH swelling AACG eye, but a relatively large decrease in RNFLT.

Figure 3 shows the RNFLT and BMO-MRW results of representative cases in the ONH swelling AACG and non-ONH swelling AACG groups.

## Discussion

In this study, we compared the ONH structure in the AACG, OAG, and healthy groups and found that the ONH of AACG was different from that of OAG. In particular, AACG with ONH swelling showed significant differences from non-ONH swelling.

Short-term high IOP can induce optic nerve ischemia and ONH swelling, and cause non-glaucomatous optic neuropathy such as non-arteritic anterior ischemic optic neuropathy (NAION)^4–6^ and similar forms have been reported in AACG^2,3,9,10^. These non-glaucomatous ONH changes have different ONH morphology compared to glaucomatous optic neuropathy^11,12^. In view of this, it has been postulated that there might be differences in the ONH structure between AACG and chronic glaucoma represented by OAG, and several studies have been conducted to evaluate the ONH structure in patients with AACG^2,13–16^. Shen et al.^13^ investigated the cup-to disc ratio and neuroretinal rim area in patients with acute primary angle-closure and found that the pattern of optic nerve damage in acute primary angle-closure was different from that of OAG. In addition, Shen et al.^17^ found that circumpapillary vessel density in patients with normal-tension glaucoma was significantly lower than that in patients with angle-closure glaucoma using optical coherence tomography angiography.

Recently, ONH structural analysis was conducted using SD-OCT. Among them, BMO-based ONH parameters were introduced, which are a useful parameter for diagnosing glaucoma^18,19^. BMO-MRW is the minimum distance from the inner opening of the Bruch’s membrane to the internal limiting membrane, and has shown superior glaucoma diagnostic power compared to previous ONH parameters^20^. Moreover, BMO-MRA has shown excellent diagnostic power in glaucoma regardless of the optic disc size^21^. To confirm that these BMO-based parameters are useful in diagnosing glaucoma, several studies have been conducted comparing glaucoma with non-glaucomatous RNFL defects, commonly represented by NAION and branch retinal vein occlusion (BRVO). In previous studies, RNFLT was matched and BMO-based parameters were compared, and BRVO and NAION showed significantly higher BMO-MRW and BMO-MRA than OAG^22,23^. In this study, the RNFLT of AACG and OAG were matched, and BMO-MRW and BMO-MRA were compared between the groups.

RNFLT showed no difference between OAG and AACG except for the inferior-temporal sector because the global RNFLT of OAG and AACG was matched at the research proposal steps. The difference in the inferior temporal sector of the RNFLT in the two glaucoma groups may be related to the fact that the inferior temporal sector of the RNFL is mainly affected by the progression of OAG^24–26^, and this result suggests that there is a difference in the mechanism of optic nerve damage in OAG and AACG.

Although the RNFLT showed similar glaucomatous damage between the OAG and AACG groups, the BMO-MRW and BMO-MRA were significantly higher in AACG than in OAG in the global and all sectors. Significant differences in BMO-based parameters between the two glaucoma groups with similar RNFL damage and BMO size also show that the mechanisms of optic nerve injury in the two groups may be different. To determine the reason for these differences, we divided the AACG group into two subgroups: those that showed optic nerve swelling and those that did not show optic nerve swelling at the onset of AACG.

Comparing the two subgroups of AACG with and without ONH swelling, global and most sectors of RNFLT were significantly lower in the ONH swelling AACG than in the non-ONH swelling AACG subgroup. In contrast, BMO-based parameters showed no significant differences between the two groups. In other words, the ONH swelling AACG group had relatively preserved BMO-based parameters compared to RNFL thinning, which is thought to be the reason why AACG had higher BMO-based parameters than OAG with the same RNFLT. The characteristic structure of the ONH of the ONH swelling AACG is consistent with the results of ONH analysis of non-glaucomatous RNFL defects, such as NAION and BRVO^22,23^. AACG with ONH swelling is considered to have a different optic nerve injury mechanism, AACG, and OAG without ONH swelling.

A sudden increase in IOP may cause condensation of the neuroretinal rim, prelaminar tissue, and deformation of the lamina cribrosa, which can cause damage to the optic nerve^27^. In addition, these changes result in a reduction of the perfusion pressure on the optic nerve and ONH swelling^28^. ONH swelling induces peripapillary capillary compression and ONH ischemia. This vicious cycle is formed when these effects occur repeatedly. This mechanism, similar to NAION, is thought to be the main mechanism responsible for optic nerve damage in AACG with ONH swelling. When RNFL thinning occurs with the above mechanism, the decrease in BMO-based parameters is relatively small compared with RNFL thinning. This is different from OAG, in which the BMO-based parameter and RNFLT simultaneously decrease owing to the continuous influence of intraocular pressure.

A previous study reported that the RNFLT decreased in AACG, but there was no change in ONH parameters, such as the cup-to-disc ratio and neuroretinal rim area^16^. The discrepancy between these ONH parameters and RNFLT could be explained by reactive gliosis which replaced lost ONH tissue^15,29–31^.

Our study has some limitations. First, all study subjects were Asian, and the ONH structure of the AACG may differ in other study populations. Second, there may have been measurement errors when calculating the BMO-MRA. For example, there may have been errors in measuring angle θ which is the angle between the BMO-MRW and BMO plane at the BMO point, or measuring r which is the distance between the BMO centroid and each BMO point. These measurement errors affect BMO-MRA. However, because all parameters were measured and calculated based on the analysed reference point using SD-OCT, the deviation can be considered insignificant.

In conclusion, AACG and OAG with similar damage in the peripapillary RNFLT show significant and clear differences in BMO-based parameters. This suggests that these two diseases are driven by different mechanisms of optic nerve damage.

## Methods

This retrospective, cross-sectional study adhered to the Declaration of Helsinki and was approved by the institutional review board of Kangdong Sacred Heart Hospital. Informed consent was waived for the study due to its retrospective nature, which was confirmed by institutional review board of Kangdong Sacred Heart Hospital (No. 2022-07-006).

### Subjects

Medical records of patients who visited the glaucoma clinic of Kangdong Sacred Heart Hospital between January 2017 and January 2021 were retrospectively reviewed. This study included 30 eyes with OAG, 30 eyes with AACG, and 30 healthy eyes. Eyes with OAG and those with AACG were matched with healthy eyes in terms of age and optic disc size, based on the BMO area. Furthermore, eyes with OAG were matched with eyes with AACG in terms of global RNFLT.

All subjects underwent ophthalmologic examinations, including best-corrected visual acuity, automatic refraction, slit-lamp biomicroscopy, Goldmann applanation tonometry, gonioscopy, fundus photography (TRC-NW8; Topcon Medical Systems, Inc., Oakland, NJ), and SD-OCT (Spectralis HRA&OCT, software version 1.10.2.0; Heidelberg Engineering, Heidelberg, Germany). In patients with OAG and AACG, visual field tests (Humphrey Field Analyzer, HFA II; Carl Zeiss Meditec, Inc., Dublin, CA) were performed.

OAG was diagnosed in the presence of characteristic glaucomatous ONH features with corresponding glaucomatous visual field defects and an open angle on gonioscopy. Glaucomatous ONH features included neuroretinal rim notching or relative thinning and a corresponding RNFL defect. Glaucomatous visual field defects were defined as those with at least two consecutive positive results for the following three criteria in reliable visual field tests (false-positive error of < 15%, false-negative error of < 15%, and fixation loss of < 20%): (1) a cluster of three points with probabilities of < 5% on the pattern deviation map in at least one hemifield, including at least one point with a probability of < 1%; (2) a glaucoma hemifield test result outside normal limits; or (3) a pattern standard deviation outside 95% of normal limits.

AAC was defined as an angle closure on gonioscopy and intraocular pressure > 40 mmHg with characteristic acute symptoms, such as headache, eye pain, and nausea. AACG was diagnosed when RNFL thinning and visual defects were observed. Patients who already had RNFL thinning at the time of AAC were excluded. In this study, the AACG group was compared with the other groups based on the test results obtained 6 months after the onset of AACG.

The AACG group was divided into the “ONH swelling AACG” and “non-ONH swelling AACG” subgroups, according to the presence of ONH swelling at the onset of AACG. ONH swelling was defined as a characteristic edematous change of the ONH on fundus examination or fundus photography, and global RNFL > 99% of the normal reference value provided by the OCT manufacturer.

Exclusion criteria were the presence of anterior segment ocular diseases or retinal disorders affecting the ONH on fundus examination, previous ocular surgery history except cataract surgery without complications, SD-OCT image quality scores < 20 dB, and SD-OCT segmentation error in RNFL and ONH analysis. For patients with both eyes meeting the inclusion criteria, only a unilateral eye was included in the study.

### Evaluation of RNFLT

The RNFLT was measured using SD-OCT through a circular scan consisting of 768 A-scans based on a 3.5-mm in diameter peripapillary circle centered on the optic disc. The global RNFLT and RNFLT in six sectors (superior temporal, temporal, inferior temporal, inferior nasal, nasal, and superior nasal) were analyzed.

### Evaluation of BMO-MRW and BMO-MRA

**Figure 4:**
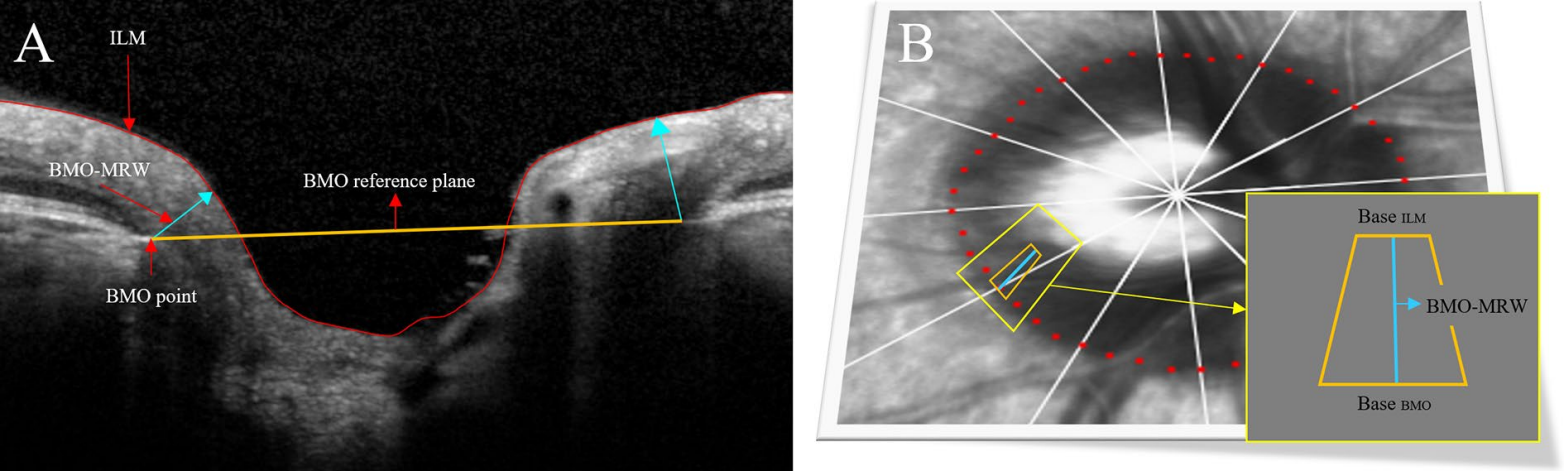
Optic nerve head analysis using spectral-domain optical coherence tomography. (**A**) Bruch’s membrane opening-minimum rim width (BMO-MRW) is defined as the shortest distance from the Bruch’s membrane opening (BMO) point to the inner limiting membrane (ILM). (**B**) Bruch’s membrane opening-minimum rim area is defined as the area of a trapezium at angle θ above the BMO plane.

BMO-MRW was defined as the shortest distance from the BMO point to the inner limiting membrane (Fig. 4). BMO points were automatically analysed using OCT. A glaucoma specialist (KIN) confirmed them, and if errors were identified, they were excluded from the study. For the measurement of BMO-MRW using SD-OCT, the BMO plane was delineated in 24 radial B-scans, each covering a 15° region centered on the ONH, and two B-scans were obtained for each region. A total of 48 measured BMO-MRWs were divided into global BMO-MRW and BMO-MRW for six sectors (superior temporal, temporal, inferior temporal, inferior nasal, nasal, and superior nasal). BMO-MRA was calculated as the total of the 48 trapezoid areas, each extending from the BMO point to the inner limiting membrane at an angle θ above the BMO plane. To calculate each trapezoid area, the height of the trapezium was considered equal to the minimum rim width, that is, BMO-MRW (W); the base of the trapezium was considered approximately equal to that of one of the BMO perimeters of 48 trapeziums, that is, 2πr/48 (r was defined as the distance between the BMO centroid and each delineated BMO point); and the top of the trapezium was calculated as 2π/48 × (r − W × cos(θ)). Therefore, the area of each trapezium was calculated using the following formula: (2πr/48 + 2π/48 × (r − W × cos(θ))) × W/2, and the delineated areas were divided into total BMO-MRA and BMO-MRA of several sectors according to BMO-MRW.

### Statistical analysis

Statistical analysis was performed using IBM SPSS ver. 25.0 (IBM Corp., Armonk, NY) and MedCalc version 9.3.7.0 (MedCalc Software, Ma Riakerke, Belgium). One-way analysis of variance with Duncan’s post hoc test was used to compare age, IOP, BMO area, global and sectorial RNFLT, BMO-MRW, and total and sectorial BMO-MRA among the three groups. Chi-square test was used to compare sex among the groups. Mann–Whitney test was used to compare each parameter between the ONH swelling AACG and non-ONH swelling AACG groups. A *P* value < 0.05 was considered statistically significant.

## Data Availability

The datasets generated during the current study are available from the corresponding author on reasonable request.
